# The Use of Index-Matched Beads in Optical Particle Counters

**DOI:** 10.6028/jres.119.029

**Published:** 2014-01-08

**Authors:** Zhishang Hu, Dean C Ripple

**Affiliations:** Center for Computational and Systems Biology, Institute of Biophysics, Chinese Academy of Sciences, Beijing, China; National Institute of Standards and Technology, Gaithersburg, MD 20899

**Keywords:** bead, flow imaging, flow microscopy, light obscuration, particles, refractive index

## Abstract

In this paper, we demonstrate the use of 2-pyridinemethanol (2P) aqueous solutions as a refractive index matching liquid. The high refractive index and low viscosity of 2P-water mixtures enables refractive index matching of beads that cannot be index matched with glycerol-water or sucrose-water solutions, such as silica beads that have the refractive index of bulk fused silica or of polymethylmethacrylate beads. Suspensions of beads in a nearly index-matching liquid are a useful tool to understand the response of particle counting instruments to particles of low optical contrast, such as aggregated protein particles. Data from flow imaging and light obscuration instruments are presented for bead diameters ranging from 6 µm to 69 µm, in a matrix liquid spanning the point of matched refractive index.

## 1. Introduction

Proper assessment of the concentration of particles in protein drugs within specified diameter limits [1,2] requires that particle counters accurately measure both particle count and diameter. However, proteinaceous particles that form by self-association of protein molecules (termed “protein particles” in this paper) are known to give particle concentrations that differ by a factor of ten or more on different types of particle counting instruments [3–5]. Protein particles have an irregular morphology, possibly fibrous or elongated structure, and low optical contrast (equivalent to a small difference in refractive index between the particle and the matrix fluid) [6,7]. Of these attributes, the low refractive index is known to cause a reduction in the reported diameter in optical counting instruments.

The two optical particle-counting methods most commonly used to measure particle concentrations in the size range from 2 µm to 200 µm are flow imaging and light obscuration. Flow imaging (FI) systems measure the projected area of particles passing through a flow cell. Light obscuration (LO) instruments measure the attenuation of a light beam caused by the combined light scattering and absorption of a particle. Both aggregated protein and the polystyrene latex (PSL) beads commonly used for instrument calibration are highly transparent, so the light attenuation is predominantly due to light scattering. Consequently, the LO instrument reports the diameter of a PSL bead that scatters the same amount of light as the actual particle. Theoretical calculations [8] and experimental results [9] indicate that the low refractive index of protein particles causes a lack of sensitivity for all of the optically based methods. Condensed, amorphous protein has a refractive index of ≈ 1.40 [10]. Aqueous solutions have a refractive index of 1.33 for nearly pure water and rising with increasing solute concentration (e.g., both protein monomer and excipients). From the difference of these values, a highly compact protein particle would have Δ*n* ≈ 0.07 as a maximum value; increased solute concentration or a high level of particle hydration or porosity would drive Δ*n* to lower values. In contrast, PSL beads in a water matrix have Δ*n* ≈ 0.25. Calculations of the response of light obscuration counters reveal that as Δ*n* increases above ≈ 0.15, the scattering strength approaches a plateau and is insensitive to further increases in Δ*n* [8]. For Δ*n* << 0.15, the scattering intensity is proportional to Δ*n*^2^. As a result, calibration of LO counters with PSL beads is primarily a problem for particles with Δ*n* < 0.15, as is the case with protein particles. Consequently, particles with small Δ*n* are reported as being undersized when measured in an instrument calibrated with PSL beads.

Table 1 presents the refractive indices (at a wavelength of 589 nm) of PSL beads and several other types of beads. The response of particle counting instruments to particles of low optical contrast can be evaluated by measuring suspensions of beads in a liquid of varying Δ*n*. Silica beads fabricated by the Stöber-Fink-Bohn (STB) process [11] have typical refractive indices in the range 1.42 to 1.43, which is readily matched by glycerol-water or sucrose-water solutions. Limitations of STB silica beads are that the refractive index of the beads varies with details of the processing and that STB silica beads are not available at diameters of ≈ 10 µm and larger. Poly(methylmethacrylate) (PMMA) and fused silica beads are available at larger sizes and have refractive indices close to the bulk-material values, but cannot be matched with common aqueous mixtures.

The ideal index-matching liquid should have a refractive index up to 1.49, be water soluble to promote ready instrument cleaning, and have a sufficiently low viscosity to allow ready clearing of air bubbles on sample mixing. Aqueous mixtures of sucrose, ethylene glycol, or glycerol have all been used as refractive index matching liquids [14], but these solutions are limited to a refractive index of approximately 1.44 for viscosities less than 60 mPa·s, or 1.46 for viscosities less than 300 mPa·s.

We examine in this paper the use of STB silica, fused silica, and PMMA beads of known size, suspended in a fluid of known refractive index, as a means of characterizing the response of a light obscuration instrument and a flow imaging instrument to particles of low optical contrast. Both 2-pyridinemethanol (2P) and glycerol were used as the high refractive-index diluent for silica beads (refractive index of ≈ 1.43 and ≈ 1.47 for different lots of beads used for this work) and PMMA (refractive index of 1.49). We demonstrate that 2P-water solutions work well at refractive indices up to 1.52 in these instrument counters. Zölls *et al.* have used sucrose solutions successfully for refractive index matching of STB silica beads in a similar study [9]. Use of 2P-water mixtures has two advantages relative to other aqueous solutions: 2P has a high refractive index (≈ 1.54) and relatively low viscosity, which allows creation of aqueous mixtures over a wide range of refractive indices with acceptable viscosities. There are two potential disadvantages to the use of 2P: the material degrades with exposure to light or long-term storage, and the liquid is slightly optically absorbing.

To prepare and use a bead standard of known diameter and concentration, beads of known size are first dispersed in water. The refractive index of water differs sufficiently from that of common bead material to enable accurate calibration of the count of the aqueous suspension against commercial PSL count standards. The bead suspension is then mixed with filtered, water-miscible solvents that increase the refractive index of the liquid and reduce the optical contrast between the beads and liquid in a controlled manner.

Because of the variability of refractive index of silica beads, the refractive index must be measured for each lot of beads. A series of suspensions with fixed bead concentration and known liquid refractive index, spanning the expected refractive index of the bead, is prepared first. Then the relative optical contrast of the beads can be measured in one of two ways. Zölls *et al.* [9] measured the apparent absorbance of the beads in a plate reader, which is highly insensitive to mixture viscosity and particle sedimentation. The minimum in absorbance corresponds to the point of refractive index matching. We report a different method in this paper, in which the series of suspensions is measured in a light obscuration counter. The light obscuration counter should have diameter bins adjusted to appropriately match the apparent particle size distribution of the silica beads. The minimum in the reported diameter as a function of refractive index corresponds to the point of refractive index matching. Measurement of the contrast by use of standard UV-VIS absorption spectrometers is not practical due to settling of the beads during the measurement.

PMMA beads are available at larger sizes than silica beads, have good optical homogeneity, and have a predictable refractive index. The refractive index of PMMA is appreciably lower than that of PSL (1.49 versus 1.59) [12], enabling the mixing of index-matched aqueous bead solutions.

## 2. Materials and Methods

### 2.1 Materials

Silica beads of reported 6.1 µm diameter were obtained from Bangs Laboratories (Fishers, IN), and of reported 10.3 µm and 25.0 µm diameter from Corpuscular, Inc. (Cold Spring, NY).^1^ Bead sizes were verified by measurement of the beads suspended in water on calibrated flow imaging and light obscuration particle counters (see 2.2 Methods for instrument details). The nominal 25 µm diameter beads gave unexpectedly low diameters. Subsequently, the 10.3 µm and 25.0 µm were measured by brightfield microscopy on a calibrated optical microscope (20× magnification), resulting in measured diameters of 10.2 µm and 19.9 µm. PMMA beads of a nominal size range 63 µm to 75 µm were obtained from Cospheric (Santa Barbara, CA). Both bead types were supplied in dry form. We formed suspensions in water by adding dry beads to water, shaking vigorously for 40 s, and then sonicating for 60 s. The counts of the bead stock were determined by diluting the stock solution 10:1 and then measuring the diluted sample on a calibrated light obscuration instrument.

For the matrix fluids, ultrafiltered water (Barnstead NANOpure system, Dubuque, IA) was combined with either 2P (98 % nominal purity from Sigma Aldrich, St. Louis, MO) or glycerol (ACS Reagent Grade, from Mallinckrodt Baker, Phillipsburg, NJ). Prior to use, the solutions were filtered through a 0.22 µm, PVDF membrane filter (Millipore GV syringe filter). Particle suspensions were stored in polyethylene sample bottles that had been precleaned by agitation in 0.1 % mass concentration sodium dodecyl sulfate solution, followed by two rinses with ultrafiltered water. Lack of appreciable particles in the water and other solutions was confirmed by measurement by light obscuration and flow imaging. The refractive index of prepared mixtures was measured at the sodium D line (589 nm wavelength), using an Abbe refractometer that had been calibrated using ultrafiltered water and NIST Standard Reference Material 1922, a mineral oil with known refractive index.

### 2.2 Methods

The prepared samples were measured by light obscuration and by flow imaging. The light obscuration particle counter (PAMAS model SVSS-C with a HCB-LD-25/25 sensor head, Leonberg, Germany) had a size range from 1 µm to 200 µm diameter. To minimize the effects of particle settling during the span of each measurement, the measurement volume was only 0.4 mL and the sampling needle was positioned halfway down into the bead suspension.

For the flow imaging results, a Micro-Flow Imaging DPA-4200 (ProteinSimple, San Jose, CA; settings: 100 µm thick flow cell, at “SP3” optical configuration) was used. Because the silica beads settle much more rapidly than PSL beads, the measurement protocol was modified. The instrument illumination was optimized with a particle-free sample of the same liquid used to suspend the particles. A particle-laden sample of 0.42 mL was then added to the instrument inlet, and the measurement commenced without further priming. As a result, the measured volume consisted of the particle-free liquid in the inlet tube between the counting chamber and the inlet, and the added particle-laden sample. The measurement volume was adjusted so that the measurement terminated with no more than 10 µL of liquid remaining above the counting chamber. The particle concentration results were multiplied by a constant factor to account for the volume of particle-free liquid included in the measurement. Although this method accounts for particle settling, there were still difficulties with beads adhering to the sides of the inlet tubing.

For each bead type and matrix-fluid refractive index, the particle size distribution was inspected, and upper and lower diameter limits were adjusted to exclude any extraneous particles that were clearly not beads. This adjustment can be done in the data analysis step for flow imaging, but for light obscuration, the instrument settings were adjusted. The reported concentration is the number of particles per unit volume between these diameter limits.

Both light obscuration and flow imaging counters were checked for correct operation by measuring nearly particle-free filtered water as a blank and by measuring commercial PSL bead count standards (ThermoScientific, Fremont, CA).

## 3. Results

To study how indicated diameter varied with the refractive index difference Δ*n*, 6.1 µm, 10.2 µm, and 19.9 µm diameter silica beads and 69 µm PMMA beads were measured by flow imaging and light obscuration. The beads were immersed in either glycerol-water or 2P-water mixtures of varying refractive index. Data were obtained for particle diameter and, where not prevented by fast sedimentation, bead concentration.

Both light obscuration and flow imaging instruments gave results for the particle diameter that agreed with manufacturer’s value for 6.1 µm, 10.2 µm, and 69 um beads, and with the brightfield microscopy value for 19.9 µm beads, when the particles were immersed in water (data not shown).

**Fig. 1:**
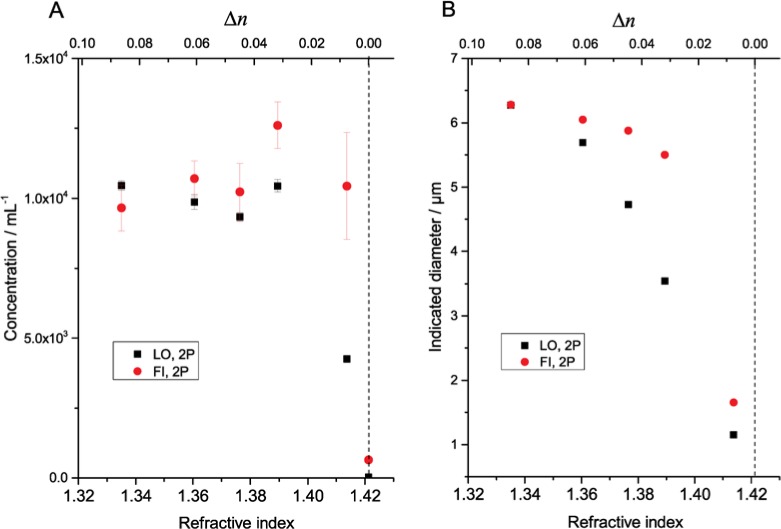
Measured concentration (A) and indicated diameter (B) of silica beads (6.1 µm diameter) suspended in 2P-water mixtures of varying refractive index. Error bars indicate standard deviations of three repeat measurements of single samples. Error bars for indicated diameter are smaller than the symbol size. The dashed vertical line indicates the approximate point of refractive index match between the beads and the matrix liquid.

**Fig. 2:**
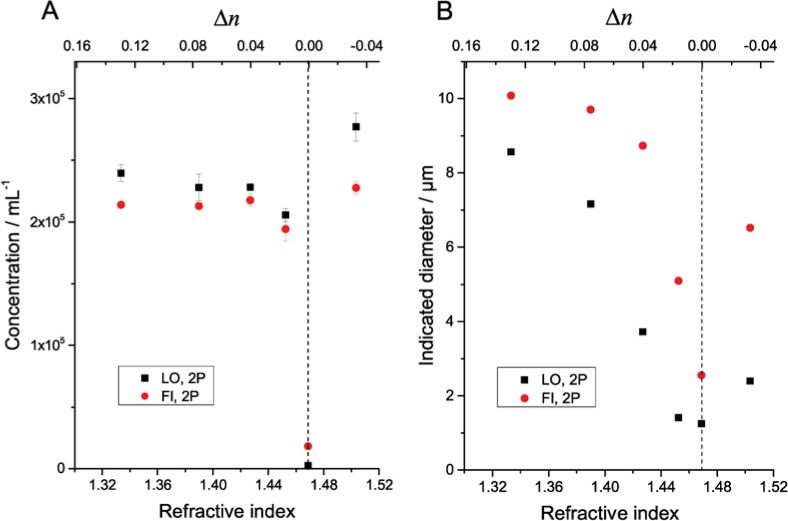
Measured concentration (A) and indicated diameter (B) of silica beads (10.2 µm diameter) suspended in 2P-water mixtures of varying refractive index. Error bars indicate standard deviations of three repeat measurements of single samples. Error bars for indicated diameter are smaller than the symbol size. The dashed vertical line indicates the approximate point of refractive index match between the beads and the matrix liquid.

**Fig. 3:**
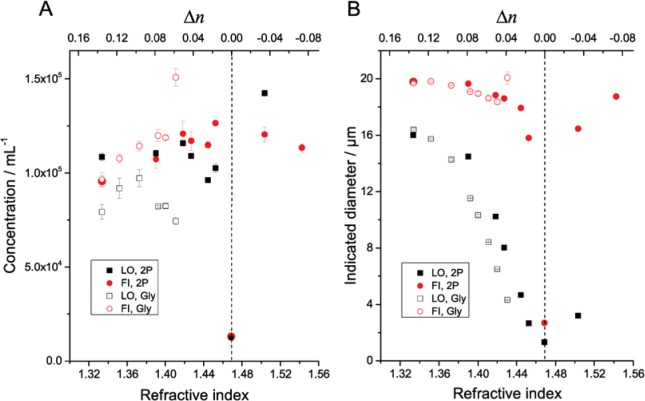
Measured concentration (A) and indicated diameter (B) of silica beads (19.9 µm diameter) suspended in 2P-water and glycerol-water mixtures of varying refractive index. Error bars indicate standard deviations of three repeat measurements of single samples. Error bars for indicated diameter are smaller than the symbol size. The dashed vertical line indicates the approximate point of refractive index match between the beads and the matrix liquid.

**Fig. 4:**
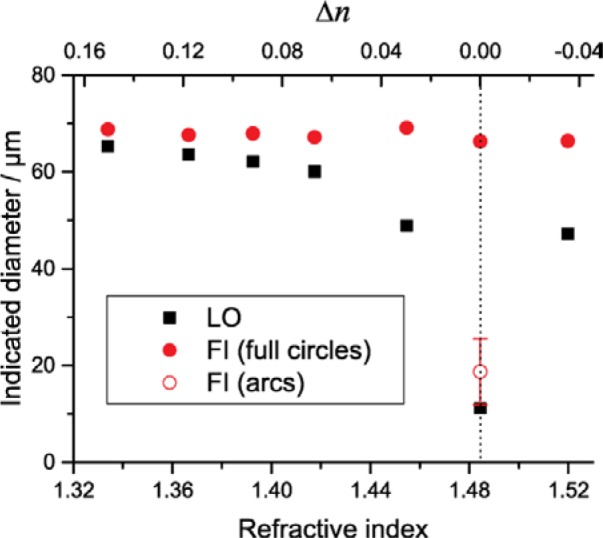
Indicated diameter of PMMA beads (≈ 69 µm diameter) suspended in 2P-water mixtures of varying refractive index. Standard deviations of repeat measurements are smaller than the symbol size. At the highest refractive index (*n* = 1.484, Δ*n* ≈ 0.007), flow imaging results were interpreted either as fully filled circles (filled red circle) or as arcs (open red circle). The dashed vertical line indicates the point of refractive index match between the beads and the matrix liquid, based on literature values for PMMA.

Figures 1–4 illustrate the undersizing of beads by light obscuration and flow imaging for small Δ*n* for silica and PMMA beads ranging in diameter from 6.1 µm to 69 µm. (In the figures, light obscuration and flow imaging data sets are designated LO and FI, respectively. 2P-water and glycerol-water mixtures are designated 2P and Gly.) The dashed vertical lines indicating Δ*n* = 0 is only approximately determined for the silica beads; additional data on solutions with small Δ*n* would be necessary to find the index-matching point accurately. As Δ*n* approaches zero, the light-obscuration diameter of each bead drops. The number of particles, however, remains relatively constant. Once Δ*n* becomes small enough that the particle size distribution of the beads is near the lower limit of the instrument, the particle count drops precipitously. Note that the reported diameter never drops below the lower threshold of the particle counter (1 µm for the counter used here): at very small Δ*n* when no peak is observed in the particle size distribution, the particle counter only reports particles having larger than average scattering.

The refractive index of the fluid where the beads have the smallest indicated diameter (corresponding to Δ*n* ≈ 0) varied substantially among the measured silica bead lots. For the smaller 6.1 µm beads, the minimum diameter occurs at a fluid refractive index of ≈ 1.43, consistent with the refractive index expected for amorphous silica beads fabricated by the Stöber-Fink-Bohn process [11]. For the larger 10.2 µm and 19.9 µm beads, the minimum diameter occurred at a fluid refractive index of ≈ 1.47, which is much closer to the refractive index expected of bulk silica (≈ 1.456) [13].

For the flow imaging results, the reported diameter of the 6.1 µm and 10.2 µm beads drops as Δ*n* approaches zero, but not as rapidly as with light obscuration. The reason for the decrease in diameter of the 6.1 µm beads is that the optical resolution of the instrument is approximately 2 µm to 3 µm: as Δ*n* decreases, the optical image of a particle of diameter near the diffraction limit retains the same size and shape, but with reduced contrast. As the contrast drops, the particle recognition algorithm identifies the particle boundaries as being closer and closer to the particle center.

**Fig. 5:**
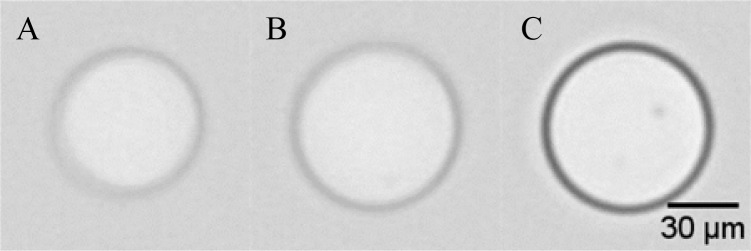
Three flow-imaging micrographs of PMMA beads (≈ 69 µm diameter) suspended in a 2P-water mixture (Δ*n* ≈ 0.007). Bead (A) was identified by automated software as only an arc; beads (B) and (C) were identified as full, filled circles.

**Fig. 6:**
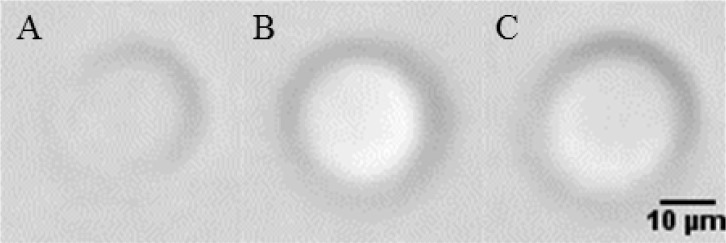
Three flow-imaging micrographs of silica beads (≈ 20 µm diameter) suspended in a 2P-water mixture (refractive index of matrix liquid = 1.4686). Bead (A) was identified by automated software as only a partial circle; beads (B) and (C) were identified as full, filled circles.

For the large 69 µm PMMA beads, the diameters reported by flow imaging change less than 4 % until | Δ*n* | < 0.01. At a value of Δ*n* = 0.007, some beads are correctly identified as particles of nearly correct diameter (shown as the filled red symbol at the right of Fig. 4), whereas other beads have greatly reduced effective diameters (shown as the open red symbol at the right of Fig. 4). To illustrate this effect, Fig. 5 shows three images of different PMMA beads, with diameters of 63 µm, 72 µm, and 72 µm for beads (A) through (C), respectively. Beads (B) and (C) are correctly sized by the flow imaging system. Bead (A), however, is reported as having an effective diameter of only 26 µm, compared to an actual diameter of 63 µm. Bead (A) is undersized because the particle identification algorithm recognizes only a portion of the bead outline; thus, the particle is seen as an arc, rather than as a disc of identified boundary. For the 19.9 µm beads, the effect is not as dramatic (see Fig. 6), but many beads were identified by the software as only partial circles.

In addition to the large drop in apparent concentration near Δ*n* ≈ 0, there were smaller variations in concentration with refractive index at larger values of | Δ*n* |, with an overall trend in particle concentration increasing with higher refractive index. The cause of this trend is not known.

For the 19.9 µm silica beads, measurements were obtained using both glycerol-water and 2P-water mixtures. The apparent diameter versus refractive index values for the two mixtures appear to be slightly shifted, by the equivalent of ≈ 0.02 in refractive index. The cause of this shift is unknown, but may be a consequence of preferential adsorption of one liquid component on the bead. The apparent concentration versus refractive index agrees for the two mixtures for refractive index values below 1.38, but diverges slightly at higher refractive index values. This divergence is possibly due to problems in suspending the silica beads. Homogeneous suspension of the silica beads was difficult to obtain in the glycerol-water mixtures without entraining air bubbles for refractive indices greater than ≈ 1.41, leading to increasing scatter for the concentration measurements. It was not possible to reach the index-matching point for the 19.9 µm silica beads with a glycerol-water mixture of sufficiently low viscosity to allow measurements. The 10.2 µm silica beads and 69 µm PMMA beads have refractive indices equal to or larger than the 19.9 µm silica beads and would have presented the same difficulty in matching the bead refractive index with a glycerol-water solution.

The disadvantages of 2P-water mixtures (material degradation with exposure to light or long-term storage, and slight optical absorbance) were not significant problems in the present measurements. The refractive index of the mixtures was measured with a refractometer rather than being inferred from literature values, so shifts in refractive index due to degradation did not affect the measurements. The optical extinction also did not impair the measurements because the solutions remained largely transparent over the small-path-lengths of the flow cells used for light obscuration and flow imaging.

The stability of 2P-water mixtures was evaluated by remeasuring the refractive index of four retained samples (two samples at a 2P volume fraction of 0.62 and two samples at volume fraction 0.72, each approximately 5 mL sample volume, held in 30 mL, low molecular-weight polyethylene bottles). After storage in the dark for 24 months, the refractive index of the mixtures shifted by +0.0064 ± 0.0022 (mean and standard deviation of four samples). The magnitude of this shift is small enough that 2P-water mixtures are sufficiently stable for studying instrument performance; the shift is large enough that year or longer shelf lives of 2P-water mixtures are not attainable for Δ*n* values smaller than ≈ 0.02. We also evaluated the stability of PMMA beads in the same retained samples. The diameter of nominally 70 µm PMMA beads at the initial measurement and after 24 months was (68.89 ± 0.08) µm (mean and standard error of the mean) and (69.13 ± 0.12) µm, respectively. A t-test confirmed that there was no statistically significant change in the measured diameter. We conclude that PMMA is chemically compatible with 2P.

## 4. Conclusions

The use of 2P-water mixtures enables the measurement of refractive-index matched beads over a much broader refractive index range than other aqueous matrix liquids, such as glycerol-water or sucrose-water solutions. This broader range, in turn, allows use of silica beads made from bulk silica glass or PMMA beads, which are available in larger sizes than STB-process silica beads. The low viscosity of the 2P-water mixtures ensured that particles could be resuspended without significant air bubble entrainment.

The data obtained for flow imaging and light obscuration instruments demonstrate the significant shifts in measured diameter that occur when the refractive index of the particle is close to that of the matrix liquid. A challenge for future work is to understand how the response of these instruments for a homogeneous bead sample can be related to the response for inhomogeneous particles, such as aggregated protein particles.

## Figures and Tables

**Table 1:** Refractive indices of materials commonly available as beads, at 589 nm.

Bead type	Refractive index
PSL [12]	1.5915
PMMA [12]	1.4905
Silica, STB [11]	1.38 to 1.45 max. range 1.42 to 1.43 typical
Silica, fused [13]	1.4584

## Footnotes

1 Certain commercial equipment, instruments, or materials are identified in this document. Such identification is not intended to imply recommendation or endorsement by the National Institute of Standards and Technology, nor is it intended to imply that the products identified are necessarily the best available for the purpose.
